# Transcriptomic analysis of fetal membranes reveals pathways involved in preterm birth

**DOI:** 10.1186/s12920-019-0498-3

**Published:** 2019-04-01

**Authors:** Silvana Pereyra, Claudio Sosa, Bernardo Bertoni, Rossana Sapiro

**Affiliations:** 1 Departamento de Genética, Facultad de Medicina, Universidad de la República, Av. General Flores 2125, C.P, 11800 Montevideo, Uruguay; 2 Clínica Ginecotologica “C”, Centro Hospitalario Pereira Rossell, Facultad de Medicina, Universidad de la República, Bvar. General Artigas 1590, C:P.11600, Montevideo, Uruguay; 3 Departamento de Histología y Embriología, Facultad de Medicina, Universidad de la República, Av. General Flores 2125, C.P, 11800 Montevideo, Uruguay

**Keywords:** RNA-Seq, Preterm birth, Gestational age, Biomarkers

## Abstract

**Background:**

Preterm birth (PTB), defined as infant delivery before 37 weeks of completed gestation, results from the interaction of both genetic and environmental components and constitutes a complex multifactorial syndrome. Transcriptome analysis of PTB has proven challenging because of the multiple causes of PTB and the numerous maternal and fetal gestational tissues that must interact to facilitate parturition. The transcriptome of the chorioamnion membranes at the site of rupture in PTB and term fetuses may reflect the molecular pathways of preterm labor.

**Methods:**

In this work, chorioamnion membranes from severe preterm and term fetuses were analyzed using RNA sequencing. Functional annotations and pathway analysis of differentially expressed genes were performed with the GAGE and GOSeq packages. A subset of differentially expressed genes in PTB was validated in a larger cohort using qRT-PCR and by comparing our results with genes and pathways previously reported in the literature.

**Results:**

A total of 270 genes were differentially expressed (DE): 252 were upregulated and 18 were down-regulated in severe preterm births relative to term births. Inflammatory and immunological pathways were upregulated in PTB. Both types of pathways were previously suggested to lead to PTB. Pathways that were not previously reported in PTB, such as the hemopoietic pathway, appeared upregulated in preterm membranes. A group of 18 downregulated genes discriminated between term and severe preterm cases. These genes potentially characterize a severe preterm transcriptome pattern and therefore are candidate genes for understanding the syndrome. Some of the downregulated genes are involved in the nervous system, morphogenesis (*WNT1, DLX5, PAPPA2*) and ion channel complexes (*KCNJ16, KCNB1*), making them good candidates as biomarkers of PTB.

**Conclusions:**

The identification of this DE gene pattern will help with the development of a multi-gene disease classifier. These markers were generated in an admixed South American population in which PTB has a high incidence. Since the genetic background may differentially impact different populations, it is necessary to include populations such as those from South America and Africa, which are usually excluded from high-throughput approaches. These classifiers should be compared to those in other populations to obtain a global landscape of PTB.

**Electronic supplementary material:**

The online version of this article (10.1186/s12920-019-0498-3) contains supplementary material, which is available to authorized users.

## Background

Preterm birth (PTB), defined as the delivery of an infant before 37 weeks of completed gestation, is a worldwide health problem and remains the leading cause of global perinatal morbidity and mortality [1–3]. PTB is a complex, multifactorial syndrome comprised of multiple clinical subtypes that can be defined as either idiopathic or ‘medically indicated’ (by caesarean or labor induction). Idiopathic or spontaneous PTB (sPTB) accounts for 70% of total PTB, while medically indicated PTB usually represents 30% of total PTB [4, 5]. Within the sPTB group, 45% of cases may occur without preterm rupture of membranes, while the remaining 25% are the consequence of the preterm premature rupture of membranes (PPROM) [5–8]. PTB has also been stratified according to gestational age (GA); neonates born between 24 and 33 weeks (severe PTB) are at higher risk of death and diseases later in life than moderate PTB (GA between 34 and 36 weeks). It has been speculated that PTB from different GA groups has diverse causes and/or pathological mechanisms [8]. Regardless of the PTB subtype, current therapies are not successful in prolonging time to birth once labor has been initiated [3].

PTB occurs as a result of the interaction of both genetic and environmental components, and it constitutes a complex multifactorial syndrome [3, 9]. The genetic architecture of pregnancy and PTB has proven challenging not only because of the multiple causes of PTB but also because of the numerous maternal and fetal gestational tissues that must interact to facilitate parturition [3, 10]. These tissues include the decidua, myometrium, cervix, maternal blood originating from the mother and villous placenta, fetal membranes (chorion and amnion), umbilical cord, and fetal blood originating from the fetus [5].

The main etiological factors related to PTB are inflammation, hemorrhage, activation of the maternal or fetal hypothalamic-pituitary axis, immune dysregulation, distension of the myometrium and cervical insufficiency [1, 3, 9, 11]. All these processes have diverse and distinctive ways of initiating labor but may share a common pathway that ends in the release of mediators that stimulate myometrial contraction, degradation of extracellular matrix components, inflammation and apoptosis. Consequently, these processes promote membrane rupture, cervical ripening, and uterine emptying, resulting in PTB [1, 11]. Studies of the transcriptomes of these tissues can help with the development of a molecular landscape of preterm labor and improve understanding of the physiology and pathology of term and preterm parturition. Specifically, study of the transcriptome of the membranes at the site of rupture in PTB may indicate shared genes of those pathways.

RNA sequencing (RNA-Seq) is a potent technology for transcriptome analysis that allows for a comprehensive characterization of gene expression [12]. The published RNA-Seq studies on human labor have been restrained to normal term pregnancies and confined to the placenta at different GAs [13–15]. More placental gene expression data are available from experiments based on microarrays [16], but most of these are concentrated on preeclampsia [13].

As mentioned, GA determines the diverse pathological mechanisms of PTB [8]. Genetic background seems to play a more relevant role in severe PTB neonates than in moderate PTB [17]. Modifications to chorioamniotic expression that end in severe PTB should be more drastic than those ending in term delivery or even moderate PTB. Therefore, we decided to focus on the transcriptome of severe PTB chorioamniotic tissues in an attempt to find DE genes with more biological significance. To validate the principal pathways found in this study, our results were compared with data previously reported in the literature.

Based on our previous work in the field [17–20] and the current status of knowledge of term and preterm labor [21–26], we anticipate modifications in inflammatory pathways in the preterm transcriptome compared to those in the term transcriptome. Nevertheless, the ultimate goal of the study is to obtain a PTB expression signature.

## Methods

### Patient recruitment

Controls and cases were term and severe preterm deliveries from unrelated offspring of women receiving obstetrical care at the Pereira Rossell Hospital Center, Montevideo, Uruguay. Preterm tissues were collected immediately after labor from pregnancies complicated by birth before 33 weeks of gestational age (GA). Term chorioamnion tissues were obtained from uncomplicated pregnancies delivered after 37 weeks of GA. Multiple gestations, fetal anomalies, eclampsia and C-surgery delivery were excluded in both groups, as well as medically indicated PTB. Due to the high incidence of premature rupture of membranes (PROM) [6, 17], we did not exclude PROM from either cases or controls, and instead we used PROM as a cofactor in the statistical analyses (see below).

Maternal demographic characteristics were collected through questionnaires filled out by the mothers after delivery. Clinical and obstetric data were obtained from the Perinatal Information System, which consists of basic perinatal clinical records developed by the Latin American Center for Perinatology (CLAP) from WHO/PAHO [27].

### Chorioamnion tissue collection, RNA extraction, and sequencing

Samples were collected within 30 min post-delivery. The amnion and chorion were obtained from the extraplacental membranes (reflected membranes), which provide a purer source of the fetal membranes. A 1 cm^2^ portion of chorioamniotic membrane surrounding the exact place of membrane rupture for each subject was collected following the procedure described by Nhan-Chang et al. [24] and immediately frozen in liquid nitrogen or submerged in RNAlater solution (Qiagen, Hilden-Germany). The amnion-chorion was processed together. All samples were later stored at − 80 °C until laboratory procedures were performed. Samples were ground into a fine powder in liquid nitrogen with a precooled pestle and mortar and subjected to RNA extraction.

Total RNA was extracted from each sample using a TRIzol® (Invitrogen, Carlsbad, California, USA) RNA extraction protocol that produces messenger RNA-enriched purification. The quality and concentration of RNA products were determined by UV-absorbance spectrophotometry (Nanodrop Technologies Inc., Wilmington, DE, USA). The integrity of the RNA molecules was checked using a 2100 Bioanalyzer platform (Agilent Technologies, Santa Clara, California, USA). We selected RNA samples from eight tissues (4 cases and 4 controls) for paired-end sequencing that were matched based on the mother’s age, fetus sex and socioeconomic status and the lack of other medical complications, such as preeclampsia or intrauterine growth restriction (IUGR). All samples had RNA integrity numbers of 8 or higher. RNA samples were shipped to Macrogen Inc. (Seoul, South Korea) under the recommended RNA submission conditions, simultaneously, to avoid batch effects [28]. Upon arrival, samples were further assessed for RNA integrity using a 2100 Bioanalyzer platform (Agilent Technologies). Messenger-RNA (mRNA) content was purified from total RNA with the PolyATract mRNA Isolation System II (Promega Inc., Madison, Wisconsin, USA) and copied into cDNA molecules using an Illumina TruSeq RNA Sample Preparation Kit v2. One cDNA library was constructed for each specimen; libraries were subjected to massive sequencing following the Illumina HiSeq2000 protocol (Illumina Inc. San Diego California, USA). A read was defined as a 100 bp cDNA fragment sequenced from both ends (paired-end). The data have been deposited in the Sequence Read Archive (NCBI) and are accessible through SRA Series accession number SRP139931. A subset of high-quality extracted RNAs, comprising a total of 15 term and 9 severe PTB samples, including the sequenced samples, were used to validate the results by real-time quantitative-PCR (see below).

### Mapping reads to the reference genome

The obtained reads were trimmed and clipped for quality control in Trimmomatic v0.32 [29] and checked for quality using FastQC v0.11.2 [30]. Reads were then aligned to the GRCh37 reference genome using Tophat v2.1.0 [31] and Ensembl annotations [32], as derived from Ensembl Release 75.

For data visualization purposes only, gene read counts were transformed by the regularized logarithm (*rlog*) [33]. This transformation removes the dependence of the variance on the mean and normalizes count data with respect to library size.

### Identification of differentially expressed (DE) genes and gene set enrichment analysis

HTSeq-count with the parameters m = union, s = no, and t = exon was used to produce raw read counts for the expression of each gene [34]. Differential expression analysis on the gene level was performed with the R packages DESeq2 v1.16.1 [33], edgeR v3.18.0 [35] and Cuffdiff v2.2.1 [36]. A linear model with preterm condition and PROM as a cofactor was employed to analyze case-control conditions. A complementary differential expression analysis contrasting RNAseq data from tissues that presented PROM (*n* = 3) vs. those without PROM (*n* = 5) using preterm as a cofactor was also performed. *P*-values were adjusted for multiple testing using the Benjamini and Hochberg (“BH”) approach [37] to control the false discovery rate (FDR) [32]. A gene was considered expressed if it had more than 5 aligned reads. Genes were identified as DE with the following criteria: absolute logarithm 2-fold change > 2 and FDR-adjusted *p*-value < 0.05 for multiple hypothesis testing (method  =  “BH”). With the aim of reducing false-positive hits, we required a gene to be selected with these two criteria by the three mentioned algorithms to be considered DE.

To understand the underlying biological processes, functional annotations of DE genes were performed using the GAGE and GOSeq packages of Bioconductor 3.5 [38, 39]. The GOSeq method considers the effect of selection bias in RNA-Seq data that can arise as a result of gene length differences [39]. All gene mapping was performed using the biomaRt R package [40]. We looked for enrichment via genetic associations with KEGG pathways and Gene Ontology (GO) terms; GO terms were supported by at least 3 analyzed genes. Multiple testing was adjusted using the BH approach, and enrichment was declared if the BH adjusted p-value was less than 0.05. Principal component analysis (PCA) was used to explore the efficiency of DE genes to explain preterm delivery.

Additionally, we investigated whether the identified severe preterm DE genes were previously associated with PTB. A reference list was generated by exploring available public databases and recent meta-analysis associations between those genes and PTB (years 2012 to 2018). Those data included a database of associations between SNPs and PTB [41], two recent meta-analysis of transcriptomic studies of several PTB tissues [5, 42], the previously mentioned transcriptomic study of chorioamnion [43] and publicly available gene expression data (http://www.genestation.org/analysis/gene/expression/ and [44–46]).

### Validation of RNA-Seq results by assessing gene expression via quantitative real-time PCR (qRT-PCR)

Six genes were selected based on greater absolute logarithm fold changes and higher statistical significance for the confirmation of DE gene data by qRT-PCR: interleukin 1 beta (*IL1B*), lipocalin 2 (*LCN2*), macrophage receptor with collagenous structure (*MARCO*), caspase 5 (*CASP5*), serpin family A member 1 (*SERPINA1*), and TNF superfamily member 15 (*TNFSF15*). The expression values of these genes were evaluated in the 15 term birth and 9 severe PTB samples.

Total RNA from each sample (1 μg) was used to synthesize first-strand cDNA using a Superscript II RT Reagent Kit (Invitrogen, Carlsbad, California, USA) and random primers (0.5 μg/μl). qRT-PCR amplifications were performed in a Corbett Real-time Thermocycler (Qiagen, Hilden-Germany) using the Biotools SYBR Green Kit (Biotools, Madrid, Spain). The relative expression ratio of a target gene was calculated as described in the 2-DDCt method [32].

Reaction mixtures contained 10 μl of QUANTIMIX EASY (Biotools; #10606–4153), each forward and reverse primer at 0.5 μM, 1 μl of cDNA sample, and deionized water to a final volume of 10 μl. The following conditions were used: 95 °C for 5 min and 40 cycles at 95 °C for 15 s, followed by 1 min at 63 °C. After amplification, a melting step was performed, with a rise in temperature from 72 to 90 °C with continuous acquisition of fluorescence.

A positive and a negative (non-template control) control were added to each PCR reaction. Each sample was assessed in duplicate, and the %CV between the duplicates was < 2%. All primer sequences for the validated genes were designed to span exon-exon junctions to minimize the potential of amplifying genomic DNA (Additional file 1). Amplification efficiencies of primers were within a range of 90 to 110% efficiency, and primer specificity was assessed by the presence of a single temperature dissociation peak. The glyceraldehyde-3-phosphate dehydrogenase (*GAPDH*) gene and the TATA-box protein (*TBP*) gene were chosen as the reference genes to estimate relative quantification. The geometric mean of the *GAPDH* and *TBP* genes was used as the reference. The relative gene expression was calculated using the 2^-ΔΔCt^ method with the term group as the control group [47].

### Validation of RNA seq results by comparison with previous preterm transcriptomic studies

To determine if the results presented here reflect the PTB mechanisms previously reported in the literature, a PubMed search was performed using these search terms: [preterm AND (transcriptome OR transcriptomic)]. The electronic search was performed on March 19th, 2018 with no restrictions to identify all articles related to DE genes in all gestational tissues. The results were analyzed based on 5 inclusion criteria: published in English, original research, human chorioamnion tissue samples, DE between term and PTB and candidate gene list assembled. All articles were cross-checked with database search results to find any additional available transcriptome data.

### Other statistical analysis and data visualization

Maternal demographics, clinical characteristics of the term and severe preterm study groups and qPCR real-time data were compared using Student’s t-test for between-group comparisons of continuous data. For comparisons of categorical data, the Chi-square test was used. All statistical analyses were performed using the R environment for statistical computing version 3.4.3 [19]. After multiple test correction, a *p*-value < 0.05 was used to consider statistical significance. The ggplot2 R package was used to create plots [48].

## Results

Demographic and clinical characteristics of the term and severe preterm groups are presented in Table 1. There were significant differences between cases and controls for gestational age, birth weight and the number of prenatal medical appointments, while the remaining assessed variables did not present significant differences between groups (Table 1). We selected 8 of the 24 chorioamniotic membranes to pursue RNA-Seq analysis (Additional file 2). The 8 tissues were selected and paired to match cases and controls based on the mother’s demographics, the newborn’s sex and socioeconomic status (see Additional file 2).

**Table 1:** Demographic and clinical characteristics of term and severe preterm patients

	Term birth	Severe preterm birth	
	Total (*n* = 15)	Total (*n* = 9)	*p* value^a^
Mothers age, years ± SE	24.1 ± 1.6	20.6 ± 1.0	0.146
Gestational age, weeks ± SE	39.3 ± 0.3	30.7 ± 0.9	1.9 × 10^− 10^
Education: elementary school or less, *n* (%)	9 (60.0)	3 (33.3)	0.399
Marital status, % single mother	13.3	22.2	0.472
PROM (%)	4 (26.7)	2 (22.2)	1
Preeclampsia (%)	0	1 (11.1)	0.238
IUGR (%)	0	1 (11.1)	0.238
Parity, *n* (%)			0.335
Nulliparous	5 (33.3)	4 (44.4)	
≥2	5 (33.3)	2 (22.2)	
Birth weight (g), mean ± SE	3492 ± 120	1696 ± 205	1,6 × 10^−07^
Birth sex, % female	47	55	0.472
Prenatal appointments ± SE	8.8 ± 0.8	4.3 ± 0.9	0.002

^a^Significance was assessed with Student’s t-test or Chi-square test, as applicable

PROM: Preterm rupture of membranes, IUGR: Intrauterine restriction growth, SE: Standard error

### Exploration and evaluation of overall gene expression

Genome-wide gene expression in chorioamnion tissue samples was evaluated using RNA-Seq technology for 4 term and 4 severe preterm samples. A summary of the sequencing read alignments to the reference genome is presented in Additional file 3. Illumina sequencing effectively produced large numbers of high-quality reads from all samples. On average, 93.7% of the total reads were mapped successfully. Among the aligned reads, 91.5% were mapped to unique genomic regions.

**Fig. 1:**
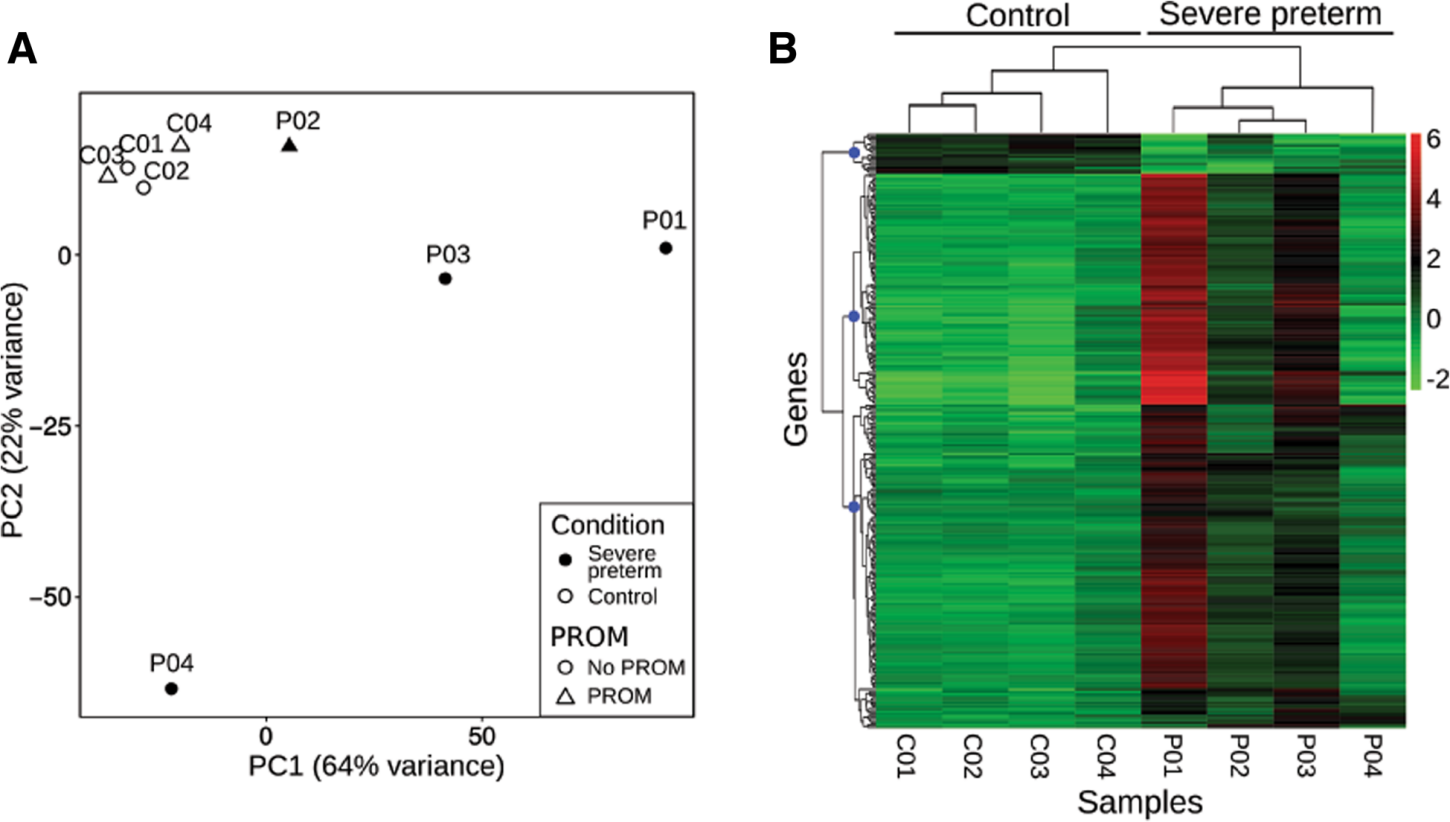
Overall gene expression of fetal membrane transcriptomes. **a** Principal component analysis (PCA) plot, denoting preterm and PROM conditions. **b** Heatmap of regularized log (rlog) normalized values of expression levels and hierarchical clustering for the 270 DE genes (FDR < 0.05 and absolute logarithm fold change > 2). The hierarchical clustering dendrogram and the condition of each sample are denoted at the top. Rlog values are coded on the red-to-green scale (high expression: red; low expression: green). Blue dots on the gene’s dendrogram nodes represent 3 major groups according to gene expression patterns. In sample names, the prefix P is used for preterms and C for controls

A PCA was performed on the gene expression rlog transformed values (Fig. 1a). A high portion of the overall variance was accounted for by the first two principal components of this model (PC1, 64%; PC2, 22%). The PCA showed term samples as a clearly packed group, while severe preterm samples showed greater variability.

A total of 270 genes were DE simultaneously according to the 3 methods employed (FDR < 0.05 and absolute logarithm fold change ≥2) (Additional file 4). Among the DE genes, 252 were upregulated and 18 were downregulated in severe preterm births relative to term births. The full list of DE genes detected (FDR < 0.05 and absolute log fold change > 2) can be found in Additional file 5a. Additional file 5 also includes references to previous studies that associated PTB with the DE genes found in this work (Additional file 5d). Approximately 50% of DE genes were previously reported to be associated with preterm labor.

**Fig. 2:**
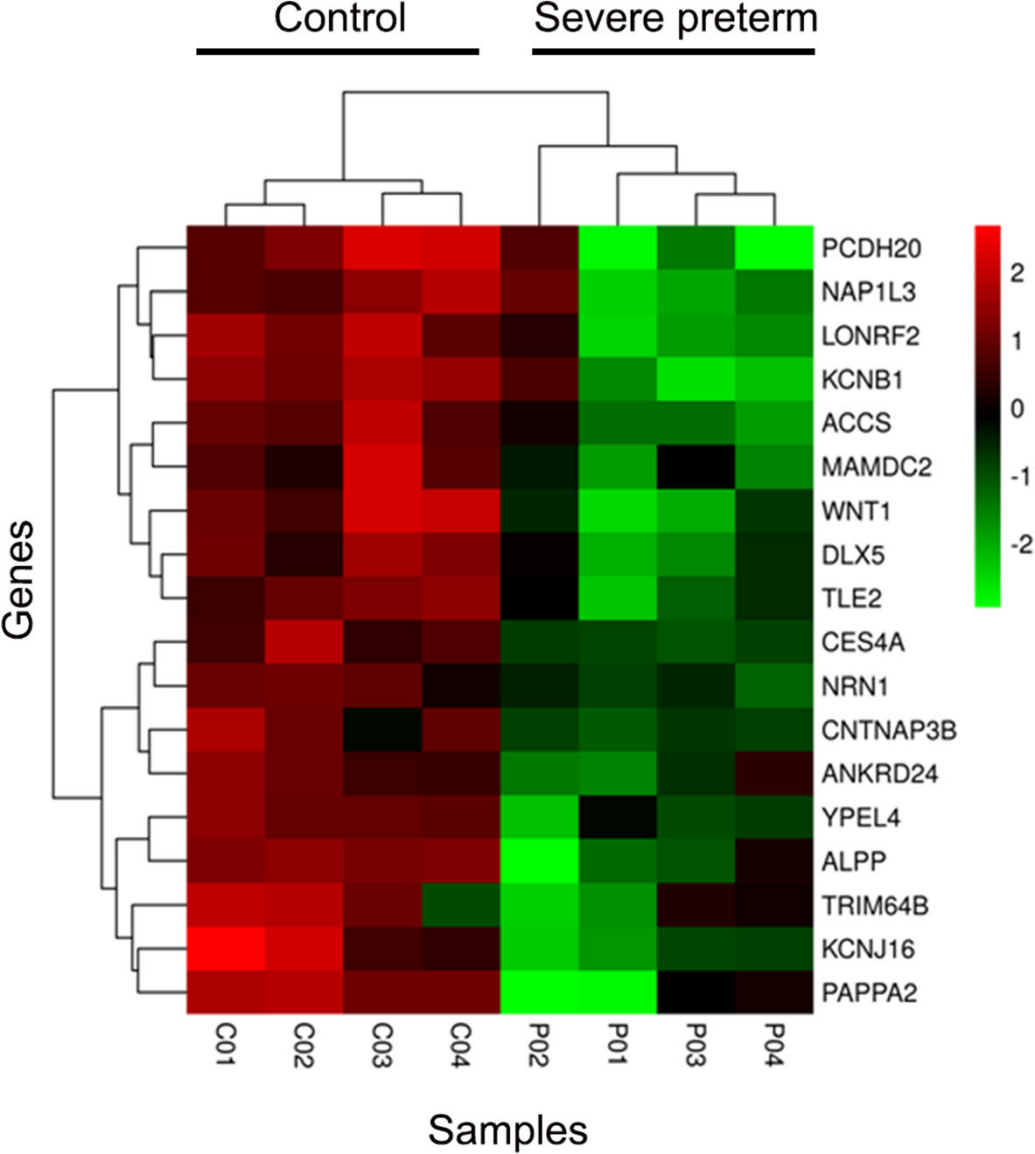
Gene expression levels of the 18 downregulated genes. Heatmap of regularized log (rlog) normalized values of expression levels and hierarchical clustering for the 18 downregulated DE genes. Gene symbols are indicated to the right. Rlog values are coded on the red-to-green scale (high expression: red; low expression: green). In sample names, the suffix P is used for preterms and C for controls

When visualizing the rlog transformed read counts of the 270 significantly DE genes, control samples had a homogenous expression pattern (Fig. 1b). Notably, several of the DE genes were associated with immune-related functions such as interleukin 24, CXC motif chemokine ligands and tumor necrosis factor signaling pathways. A hierarchical clustering analysis clustered genes in 3 groups. Remarkably, one of the clusters was a group of 18 genes that had a notably discordant expression pattern for term and severe preterm samples. This group consisted of the aforementioned 18 downregulated genes (Fig. 2), which potentially characterize a severe preterm transcriptome pattern and therefore are candidate genes for understanding the syndrome. These genes included potassium voltage-gated channel subfamily members, genes involved in cell adhesion functions, placental alkaline phosphatase and a Wnt family member. This gene cluster was related to GO terms involved in the nervous system, organ morphogenesis, and ion channel complexes, but no term remained as enriched with statistical significance in the group after BH *p*-value correction.

The remaining two groups consisted of 147 and 105 genes. Mainly, these genes were related to chemokines, TNF family members, coagulation factors, and transmembrane proteins. A GO term enrichment search revealed 508 and 301 terms, respectively that were statistically significant (BH corrected *p*-value < 0.05). These terms were mostly related to immune and inflammatory responses and the immune system.

We found some overlap between the 270 DE genes and genes related to labor that were previously identified by RNA microarrays [49–52] . In total, 58 of the 270 DE genes found in this study were identified as DE between labor and no labor births (Additional file 5, b).

When analyzing whether PROM has an effect on gene expression, we found only 14 genes differentially expressed between groups with and without PROM (FDR < 0.05 and absolute log fold change > 2) (Additional file 5, c). Only 2 of those genes were also detected as DE with respect to preterm condition: KCNJ16 and TRPV6.

### Gene set enrichment analysis using gene ontology

In an effort to identify processes and pathways that could be regulated differentially between the severe preterm and term groups, we performed a Gene Set Enrichment Analysis implemented in the Goseq R package. Genes that showed an FDR < 0.05 were tested against the background set of all expressed genes with ENSEMBL annotations. In this sense, for the enrichment analysis comparing gene expression between severe preterm and term births, 270 significant genes were tested against 18,675 background genes. The significant enrichment of GO terms was tested using the Wallenius approximation and by applying multiple hypothesis testing correction (BH). Only terms with 3 or more statistically significant genes were considered.

**Fig. 3:**
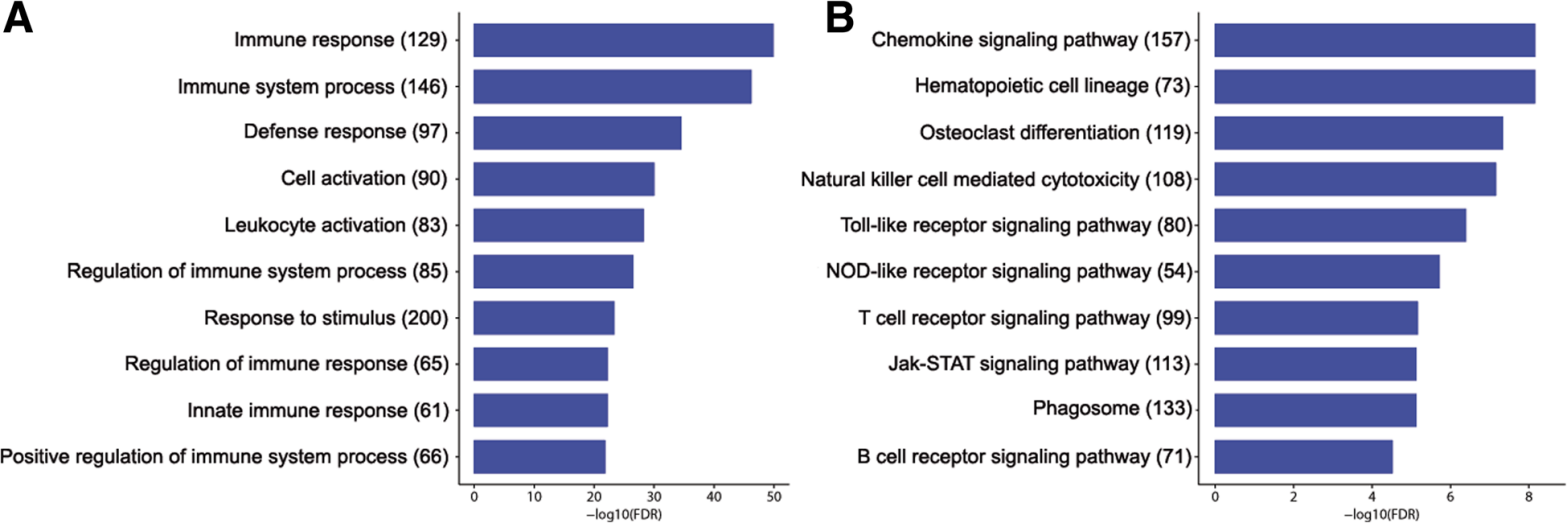
Enriched GO categories (**a**) and KEGG pathways (**b**) of genes in severe preterm birth. Enrichment analysis of Biological Process GO categories (**a**) and KEGG pathways (**b**) of DE genes in severe preterm/term comparison. The top 10 most significant terms or pathways are shown. The x-axis represents the enrichment value as the 278 logarithm of the adjusted *p*-value (FDR). The number of genes identified in each pathway is reported inside parentheses. All represented GO terms are significantly enriched, with an adjusted *p*-value < 0.01

A total of 1886 GO terms were enriched among the DE genes (FDR < 0.05), corresponding to Biological Processes (562 terms), Cellular Component (49) and Molecular Function (35) (Fig. 3a). Within the Biological Processes category, the terms *immune response*, *immune system process* and *defense response* were the most significantly enriched for DE genes. Likewise, the Cellular Component category subdivided annotated sequences into *plasma membrane*, *cell periphery,* and *cytoplasmic vesicle part*, which were the most highly represented terms. Within the category Molecular Function, the three principal groups were *signaling receptor activity*, *rec*eptor activity, and *transducer activity*.

**Fig. 4:**
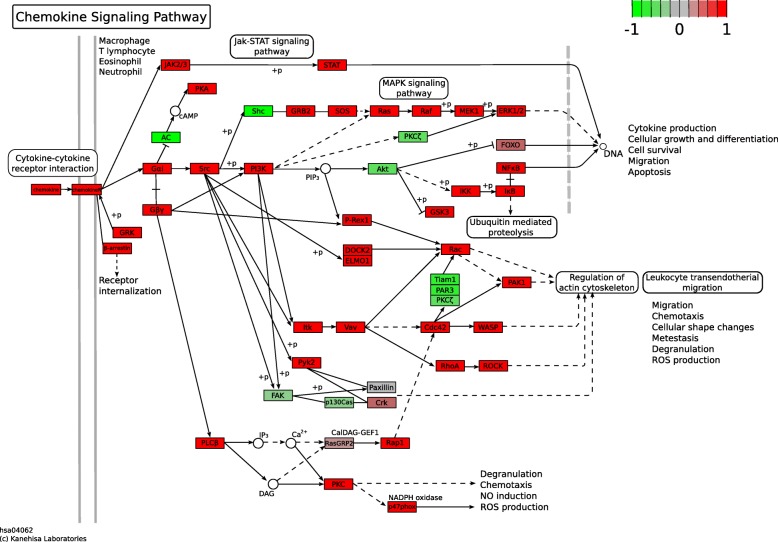
KEGG diagram of chemokine signaling pathways. Genes overexpressed in preterm birth fetal membranes are red, and downregulated genes are green

When analyzing KEGG pathways, the most significant upregulated pathways were Chemokine signaling pathway and Hematopoietic cell lineage (both with FDR = 7.038 × 10^− 09^; Fig. 4). Several relevant processes for PTB were upregulated, such as those involved in intracellular signaling: Toll-like, NOD-like, T cell and Jak-STAT signaling pathway (Fig. 3b). No statistically significant downregulated pathways were found.

### Validation of gene expression by qRT-PCR

To validate genes that were significant in the RNA-Seq analysis, six DE genes were selected—*IL1B, LCN2, MARCO, CASP5, SERPINA1* and *TNFSF15*—and their expression was assessed by qPCR. The RNA-Seq analysis showed that all genes were significantly upregulated in PTB. Additional file 6 displays the logarithm fold differences in gene expression measured by both RNA-Seq and qRT-PCR. The six genes displayed similar patterns of mRNA abundance with both methods. Five genes were significantly upregulated between severe preterm and controls when analyzing the qPCR expression data: *LCN2, MARCO* and *TNFSF15* (*p* value < 0.001) and *CASP5* and *IL1B* (*p* value < 0.05); *SERPINA1* was also upregulated, although the differences between groups did not reach statistical significance (Additional file 6).

### Validation of RNA seq results by comparison with previous preterm transcriptomic studies

The results presented in this study were compared with those previously reported in the literature. A PubMed search using the terms [preterm AND (transcriptome OR transcriptomic)] yielded 101 results. We collected all 101 abstracts and, after analyzing them, found that most studies focused on preeclampsia, placental villi tissue, and animal models or maternal or cord blood. Six studies used RNA-Seq: two focused on cord blood [53, 54], two on animal models [55, 56] and two on the transcriptome of normal labor [14, 57].

Only one study met all inclusion criteria mentioned above and included the transcriptomes of the chorion and amnion membranes; this study was included for comparison with the results obtained in this work [43]. The study used microarray technology and found 50 genes that identified the preterm labor phenotype, seven of which were DE in the current study (Table 2). The direction of gene expression changes matched between studies in all cases [43].

**Table 2:** DE genes simultaneously found in Bukowski et al. [43] and this study

Gene Symbol	Gene	HGNC ID	Change in direction
BCL2A1	BCL2 related protein A1	HGNC:991	upregulated in PTB
CCRL2	C-C motif chemokine receptor-like 2	HGNC:1612	upregulated in PTB
CHI3L1	chitinase 3 like 1	HGNC:1932	upregulated in PTB
CXCL2	C-X-C motif chemokine ligand 2	HGNC:4603	upregulated in PTB
GPR84	G protein-coupled receptor 84	HGNC:4535	upregulated in PTB
SAMSN1	SAM domain, SH3 domain and nuclear localization signals 1	HGNC:10528	upregulated in PTB
SLC16A10	solute carrier family 16 member 10	HGNC:17027	upregulated in PTB

## Discussion

Although PTB is responsible for most neonatal deaths and morbidity [58], there has been a shortage of studies that permit the creation of a distinctive expression footprint to describe its pathology and to consequently achieve improvements in its prevention, diagnosis, and treatment. To overcome this gap in knowledge, we analyzed the gene expression of the chorioamnion membranes from severe PTB and term newborns by RNA-seq.

In this study, we present a distinct transcriptome pattern of severe PTB. Our study revealed 270 DE genes between both conditions. A number of this magnitude may partially explain the lack of previous molecular signatures for PTB as well as the difficulty in identifying biomarkers and potential therapeutic interventions [4, 39]. Several pathways and genes are involved in labor, and most of them are either turned on or off during pathological conditions [4, 16, 37, 40].

It has been proposed than pregnancy is maintained by the downregulation of chemokines at the maternal-fetal interface [43]. Removal of this downregulation results in term birth, while in the case of PTB, the activation of multiple pathways of the immune system overrides this downregulation [3, 43, 55, 59–62]*.* In concordance with this idea, we found that upregulated genes were mostly related to the immune response (e.g., *IL1B*, interleukin 24, CXC motif chemokine ligands or *TNF*). Moreover, we found that severe preterm labor was significantly enriched for immune processes in comparison to term labor. Gene ontology and KEGG pathway enrichment analyses indicated that inflammatory and immune pathways were enriched in severe preterm samples (Fig. 3). These findings are in agreement with previously mentioned studies, demonstrating that preterm labor is associated with extensive immune response activation in several gestational tissues. In fact, the incidence of PTB in association with inflammation is higher earlier in pregnancy [63], so the overexpression of inflammatory and immune pathways in severe PTB is consistent with the results reported in the literature.

*IL1B* was upregulated in severe preterm births in our data. IL1B is a central mediator in the pathological process of inflammation since it can stimulate the expression and release of other labor mediators [21, 23, 64–66]. Recent evidence indicates that IL1B may be involved in labor through activation of the inflammasome [22]. The inflammasome is a multi-protein complex located in the cytoplasm of cells whose activation induces the transformation of pro-IL1B (immature form) into the active forms of IL1B via the actions of active caspase-1 (CASP1) [67–70]. NLR family pyrin domain-containing protein 3 (NLRP3) is an inflammasome recruiting pattern recognition receptor [68, 71]. The amniotic fluid concentrations of some inflammasome mediators (CASP1, IL1B, NLRP3, and IL18) are greater in women who have spontaneous preterm or term labor with intraamniotic infection/inflammation than in those without this clinical condition [22, 72–77]. We found that some inflammasome genes, including CARD domain containing 4 (*NLRC4*), caspase 5 (*CASP5*), absent in melanoma 2 (*AIM2*) and the already mentioned *NLRP3*, were upregulated in our data. Moreover, the inflammasome complex GO term (GO:0061702) was significantly enriched in this group. Taking all data together, inflammasome activation may be implicated in the induction of preterm labor. If the hypothesis that inflammasome activation contributes to PTB is true, it may have medical implications. Components of the inflammasome may be the targets of drugs that could potentially abort PTB once it is clinically suspected and before the full immunological response begins.

As can be observed in Additional file 5, several genes identified as DE in this work were previously associated with PTB. Interestingly, two of the overexpressed genes found in this study were previously proposed as biomarkers of PTB because their concentration in amniotic fluid was higher in PTB than in term labor: C-X-C motif chemokine ligand 10 (*CXCL10*) gene and matrix metallopeptidase 8 (*MMP8*) [46, 78–80]. Consequently, these biomarkers can be analyzed in amniotic fluid and provide information to prevent PTB. *CXCL10* is related to maternal anti-fetal rejection in the same way that it is related to allograft rejection [46]. In this context, *CXCL10* overexpression in PTB may lead to premature labor by triggering a fetal inflammatory response. MMP8 is known to decrease the integrity of the cervical and fetal membrane extracellular matrix [81]. MMP-8 may alter the membrane extracellular matrix and permit the passage of bacteria into the endometrium, leading to bacteria-induced PTB [82].

A small group of 18 genes clustered together discriminated between term and severe preterm cases (Fig. 2). All of the genes were downregulated. These genes potentially characterize a severe preterm transcriptome pattern and therefore are candidate genes for understanding the syndrome. None of these downregulated genes were previously proposed in the literature as preterm birth biomarkers. However, the placental alkaline phosphatase (*ALPP*) and pappalysin 1 (*PAPPA*) genes were included in a group of nine placental genes that together could predict GA in a recent pilot study [45]. *ALPP* and Pappalysin 2 (*PAPPA2*), a closely related gene to *PAPPA*, were among the 18 downregulated genes found here. Additionally, *PAPPA2*, a type of metalloproteinase with typically high gene expression in the placenta [83], was found to be DE in placenta tissue between spontaneous PTB and term birth [84].

The low number of downregulated genes made it difficult to establish statistically significant pathways: only two of the 18 clustered genes were included in the same KEGG pathway. The Wnt family member 1 (*WNT1*) and distal-less homeobox 5 (*DLX5*) genes belong to the pathway: “Signaling pathways regulating pluripotency of stem cells” (hsa04550). In term births, expression of this pathway increases at the end of pregnancy [85]. The *WNT1* gene belongs to the WNT signaling pathway, which is involved in cell proliferation, development, and tumor progression. WNT signaling pathway inhibition could contribute to miscarriages and PTB by decreasing trophoblast proliferation and invasion [86]. Notably, the *WNT2* gene, which is typically overexpressed in placenta, was not DE in our data. *DLX5* is a member of a homeobox transcription factor gene family. Homeobox genes regulate embryonic as well as placental development [87]. They are widely expressed in the human placenta. A potential role for DLX5 may be related to neurobehavioral development and to the regulation of stem cell function, both of which are important for normal placental development [87]. Additionally, *DLX5* is upregulated in preeclamptic placentas, presumably due to altered methylation at the *DLX5* locus in preeclampsia that results in loss of imprinting [88]. A decrease in the expression of this gene later in gestation was found in human placenta as a response to an increase in gene body methylation over gestational age [87]. Our results indicate that *WNT1* and *DLX5* expression are lower in severe PTB fetal membranes than in term membranes. In this context, we speculate that downregulated genes may act as maturation genes that set the gestation timing.

These putative maturation genes may be very difficult to discriminate from those leading to preterm birth. A major issue in the study of PTB transcriptomes in humans is the inability to collect healthy control tissue at the same gestational age (GA) to compare with pathologic preterm tissue. Consequently, DE genes between PTB and term fetuses reflect differences in both GA and the pathological events of preterm labor. We are aware that, as a consequence of the design of our study, genes responsible for PTB and GA overlap. By comparing human sPTB and term transcriptomes with GA-matched control transcriptomes from a closely related species (macaques), Eidem et al. [89] identified a low number of distinctive, promising sPTB-specific candidate genes or genes potentially related to GA effects. The authors found similar functions for PTB and GA genes, so they speculated that the effects of sPTB and GA do not correspond to biologically distinct processes [89].

In an attempt to narrow the gene signature of PTB, DE genes were compared with labor-related genes described in previous works [49–52]. Fifty-eight of the DE genes found in this study were identified as labor-related genes, making them candidates for labor independent of GA. In the remaining group, it is probable that PTB genes overlap with those related to GA. However, whether PTB is labor that occurs too soon or represents a pathological event with a new group of expressed genes is still an open question.

The present transcriptome of chorioamnion membranes does not resolve the controversy, but it adds information that will help to complete the PTB expression scenario. On one hand, our results confirmed that immune response pathways are upregulated in PTB. On the other hand, this RNA-Seq approach allowed the identification of upregulated genes and pathways that were not previously reported to be associated with PTB (hematopoietic cell lineage pathway, osteoclast differentiation). Additionally, we found that some of the downregulated genes are involved in the nervous system, morphogenesis (*WNT1, DLX5, PAPPA2*) and ion channel complexes (potassium voltage-gated channel subfamily J member 16 *(KCNJ16)*, potassium voltage-gated channel subfamily B member 1 (*KCNB1*)). It is difficult to speculate on the importance of these genes and pathways in PTB; for example, ion channels are involved in a wide spectrum of different functions that eventually lead to labor or PTB, including implantation [90], Ca^++^ signaling [91], smooth muscle contraction [92] and inflammation [93]. Regarding the possibility that the hematopoietic cell lineage pathway is connected to PTB, it is known that the placenta produces hematopoietic stem cells (HSCs) [94, 95]. The placenta can generate HSCs and cause their expansion but not their differentiation. The placental HSC pool first appears early in gestation and then decreases while the HSC reservoir develops in the liver [95, 96]. Subsequent studies demonstrated that mid-gestation placenta also harbors a large pool of pluripotent hematopoietic stem cells that have self-renewal capacity [94, 97]. Moreover, hematopoietic progenitors were found in full-term placentas within a low percentage of lineage-committed cells [98]. Since the hematopoietic monocyte-macrophage lineage produces osteoclasts, the hematopoietic cell lineage and osteoclast differentiation pathways are connected. Although both pathways appear far removed from term labor or PTB a priori, they may represent interesting unexplored paths to preterm labor. Nevertheless, all of the mentioned genes and biological processes open new lines of research, making them good candidates as biomarkers of PTB.

A literature search to validate these genes revealed that, surprisingly, there are few high-throughput studies analyzing chorioamniotic tissues and none using RNA-Seq technology. To our knowledge, this is the first RNA-Seq study of chorioamniotic tissues in preterm and term births. When comparing the results from Bukowski et al. [43], only 7 genes are shared with our study. All of these genes may be related to PTB in one way or another: C-C motif chemokine receptor-like 2 (*CCRL2*) and C-X-C motif chemokine ligand 2 (*CXCL2*) belong to inflammatory pathways [99, 100], and G protein-coupled receptor 84 (*GPR84*) is a receptor of fatty acids that is also related to inflammation [101]. BCL2 related protein A1 (*BCL2A1*) and chitinase 3 like 1 (*CHI3L1*) were recently associated with chorioamnionitis in monkeys and humans [102]; solute carrier family 16 member 10 (*SLC16A10*) is upregulated in the mid-gestational placenta [103]; and SAM domain, SH3 domain and nuclear localization signals 1 (*SAMSN1*) is a poorly characterized gene that may be involved in the differentiation of lymphocytes B [104]. The fact that these subsets of genes overlap in both studies, which used different experimental techniques, stresses that they should be targets of further research into PTB.

One interesting finding is the homogeneity for gene expression in the control samples. After a linear reduction of the variability for more than 30,000 expressed genes, the control samples cluster together in a small group. The dispersion of the samples in the PCA analysis is lower compared to other tissue expression profiles using the same exploratory analysis [105]. In contrast, PTB chorioamniotic expression analysis revealed a more disperse group, further stressing the heterogeneity of this condition. This may be a consequence of the complexity of the syndrome characterization, as it has been previously reported in the expression of other tissues related to PTB like the myometrium [106]. In concordance, Ngo et al. [45] developed a gene expression model based on GA that predicted time until delivery for full-term but that failed for preterm deliveries. This suggests that to predict PTB the classifiers must incorporate at least part of the several outlier physiological events that may lead to PTB [45], e.g. PROM.

Premature and preterm rupture of membranes are frequent complications of birth and, more importantly, preterm birth [107]. The transcriptome profile of the chorioamnion may differ with and without PROM. To overcome this issue, we performed statistical analyses including PROM as a co-variable.

Moreover, when analyzing the differential gene expression of PROM, we did not find substantial differences between samples from newborns with and without PROM. We only detected 14 genes with DE among groups. Additionally, the PCA did not show any clustering based on this condition (Fig. 1). This low number of DE genes related to PROM is likely the consequence of our experimental design, which focused on sampling groups discriminated by the severe preterm condition while matching for the remaining variables. Additionally, when analyzing gene expression between PROM and no PROM groups, the group that presents rupture of membranes is limited in the number of samples, meaning that the results must be confirmed with a larger sample size.

Finally, this study was performed in an admixed population where PTB is frequent (approx. 10%), indicating that it is a serious health public problem. Population-specific studies are relevant, as genetic background may differently impact different populations, e.g., it is known that African American ancestry increases the risk of PTB [108–110]. Generally, there is an information gap regarding specific populations, except those coming from North America and Europe. With our literature search, we recovered only three out of 101 transcriptomic analyses based on populations from Latin American countries [46, 111, 112], stressing the fact that most studies do not include susceptible populations from this part of the continent.

All of the Latin American studies were performed with microarray analysis, meaning that the data shown in this work are the first RNA-Seq data available to contrast against future studies. Specifically, the Uruguayan population is admixed, with a predominant European contribution and Native American and African contributions of 10.4 and 5.6%, respectively [113, 114]. Common genetic variation affects expression variability [115], as well as the admixed genome structure [116, 117], stressing the necessity of population-specific transcriptome analyses or, alternatively, including diversity in high-throughput population analyses.

## Conclusions

The use of state-of-the-art RNA-Seq analytics identified previously reported genes and pathways as well as novel candidates involved in labor and PTB. The combination of up- and downregulated genes permitted the development of a multi-gene disease classifier. These markers were generated in an admixed population in which PTB has a high incidence. Further studies including the testing of these classifiers using a second and larger independent sample are needed to validate the results. Moreover, these markers should be compared to other population studies to obtain a global landscape of severe PTB.

## Additional files

Additional file 1: **Table S1.** Primers used for quantitative PCR analysis. Sequences of the primers used for qRT-PCR. (XLSX 4 kb)

Additional file 2: **Table S2.** Clinical and demographics characteristics of 4 controls and 4 patients analyzed by RNA-seq**.** Controls are indicated as C01, C02, C03 and C04 and severe preterms as P01, P02, P03 and P04. (XLSX 12 kb)

Additional file 3: **Table S3.** Alignment statistics obtained by tophat2. Number of sequenced reads, total mapped reads and uniquely mapped reads for each of the 8 sequenced samples. (XLSX 4 kb)

Additional file 4: **Figure S1.** Differentially expressed genes. Venn diagram showing the number of differentially expressed genes identified by each of the three methods employed, when considering FDR < 0.05. (TIF 906 kb)

Additional file 5: **Table S4.** a. Full list of differentially expressed genes (FDR < 0.05 and absolute logarithm log fold change > 2) detected in the severe preterm vs. term pairwise comparison. b. Subset of DE genes that were also DE between labor and no labor conditions in the literature. c. Full list of differentially expressed genes (FDR < 0.05 and absolute logarithm fold change > 2) detected in the PROM vs. no PROM comparison. d. List of severe preterm DE genes already identified as associated with PTB in available databases. References are indicated in the manuscript. (XLSX 60 kb)

Additional file 6: **Figure S2.** Validation of overall gene expression. Log2-fold changes of six DE genes measured by RNA-Seq (red) vs. qRT-PCR (blue). *IL1B*: interleukin 1 beta, *LCN2*: lipocalin 2, *MARCO*: macrophage receptor with collagenous structure, *CASP5*: caspase 5, *SERPINA1*: serpin family A member 1, and *TNFSF15*: TNF superfamily member 15. *p < 0.05; **p < 0.01; ***p < 0.001. (EPS 1243 kb)

## Abbreviations

AIM2: Absent in melanoma 2
ALPP: placental alkaline phosphatase
BCL2A1: BCL2 related protein A1
BH: Benjamini and Hochberg’s approach
CASP5: Caspase 5
CCRL2: C-C motif chemokine receptor-like 2
CHI3L1: Chitinase 3 like 1
CLAP: Latin American Center for Perinatology
CXCL10: C-X-C motif chemokine ligand 10
CXCL2: C-X-C motif chemokine ligand 2
DE: Differentially expressed
DLX5: Distal-less homeobox 5
FDR: False discovery rate
GA: Gestational age
GAPDH: Glyceraldehyde-3-phosphate dehydrogenase
GO: Gene Ontology
GPR84: G protein-coupled receptor 84
HSCs: Hematopoietic stem cells
IL1B: Interleukin 1 beta
IUGR: Intrauterine growth restriction
KCNB1: Potassium voltage-gated channel subfamily B member 1
KCNJ16: Potassium voltage-gated channel subfamily J member 16
LCN2: Lipocalin 2
MARCO: Macrophage receptor with collagenous structure
MMP-8: Matrix metallopeptidase 8
mRNA: Messenger-RNA
NLRC4: NLR Family, caspase recruitment domain-containing 4
NLRP3: NLR Family, pyrin domain-containing 3
PAPPA: Pappalysin 1
PAPPA2: Pappalysin 2
PCA: Principal Components Analysis
PROM: Premature rupture of membranes
PTB: Preterm birth
qRT-PCR: Quantitative real-time PCR
rlog: Regularized logarithm
RNA-Seq: RNA sequencing
SAMSN1: SAM domain, SH3 domain and nuclear localization signals 1
SERPINA1: Serpin family A member 1
SLC16A10: Solute carrier family 16 member 10
sPTB: Spontaneous PTB
TBP: TATA-box protein
TNFSF15: TNF superfamily member 15
TRPV6: Transient receptor potential cation channel subfamily V member 6
WNT1: Wnt family member 1

## References

[1] Behrman RE, Stith BA (2007). Institute of Medicine Committee on understanding premature birth and assuring healthy outcomes board on health sciences outcomes: preterm birth: causes, consequences, and prevention.

[2] Liu L, Johnson HL, Cousens S, Perin J, Scott S, Lawn JE (2012). Global, regional, and national causes of child mortality: an updated systematic analysis for 2010 with time trends since 2000. Lancet.

[3] Romero R, Dey SK, Fisher SJ (2014). Preterm Labor: One Syndrome, Many Causes. Science.

[4] Myatt L, Eschenbach DA, Lye SJ, Mesiano S, Murtha AP, Williams SM (2012). A Standardized Template for Clinical Studies in Preterm Birth. Reprod Sci.

[5] Eidem HR, Ackerman WE, McGary KL, Abbot P, Rokas A (2015). Gestational tissue transcriptomics in term and preterm human pregnancies: a systematic review and meta-analysis. BMC Med Genomics.

[6] Vogel JP, Chawanpaiboon S, Moller A-B, Watananirun K, Bonet M, Lumbiganon P. The global epidemiology of preterm birth. Best Pract Res Clin Obstet Gynaecol. 2018. 10.1016/j.bpobgyn.2018.04.003. 10.1016/j.bpobgyn.2018.04.003 29779863

[7] Goldenberg RL, Culhane JF, Iams JD, Romero R. Epidemiology and causes of preterm birth. Lancet. 2008;371. 10.1016/S0140-6736(08)60074-4. 10.1016/S0140-6736(08)60074-4 PMC7134569 18177778

[8] Moutquin J (2003). Classification and heterogeneity of preterm birth. BJOG.

[9] Henderson JJ, McWilliam OA, Newnham JP, Pennell CE (2012). Preterm birth aetiology 2004–2008. Maternal factors associated with three phenotypes: spontaneous preterm labour, preterm pre-labour rupture of membranes and medically indicated preterm birth. J Matern Fetal Neonatal Med.

[10] Iams JD (2017). Preterm birth categories–labels with consequences. Am J Obstet Gynecol.

[11] Monangi NK, Brockway HM, Zhang G, Muglia LJ, House M (2015). The genetics of preterm birth: Progress and promise. Semin Perinatol.

[12] Kukurba KR, Montgomery SB (2015). RNA Sequencing and Analysis. Cold Spring Harb Protoc.

[13] Sõber S, Reiman M, Kikas T, Rull K, Inno R, Vaas P (2015). Extensive shift in placental transcriptome profile in preeclampsia and placental origin of adverse pregnancy outcomes. Sci Rep.

[14] Kim J, Zhao K, Jiang P, Lu Z-X, Wang J, Murray JC (2012). Transcriptome landscape of the human placenta. BMC Genomics.

[15] Mikheev AM, Nabekura T, Kaddoumi A, Bammler TK, Govindarajan R, Hebert MF (2008). Profiling gene expression in human placentae of different gestational ages: an OPRU Network and UW SCOR Study. Reprod Sci.

[16] Buckberry S, Bianco-Miotto T, Bent SJ, Dekker GA, Roberts CT (2014). Integrative transcriptome meta-analysis reveals widespread sex-biased gene expression at the human fetal-maternal interface. Mol Hum Reprod.

[17] Rey G, Skowronek F, Alciaturi J, Alonso J, Bertoni B, Sapiro R (2008). Toll receptor 4 Asp299Gly polymorphism and its association with preterm birth and premature rupture of membranes in a South American population. Mol Hum Reprod.

[18] Pereyra S, Velazquez T, Bertoni B, Sapiro R (2012). Rapid multiplex high resolution melting method to analyze inflammatory related SNPs in preterm birth. BMC Res Notes.

[19] Rey G, Pereyra S, Velazquez T, Grasso D, Alonso J, Bertoni B, et al. The effect of inflammation on preterm birth. In: Preterm Birth-Mother and Child. InTech; 2012.

[20] Pereyra S, Bertoni B, Sapiro R (2016). Interactions between environmental factors and maternal–fetal genetic variations: strategies to elucidate risks of preterm birth. Eur J Obstet Gynecol Reprod Biol.

[21] Romero R, Grivel J-C, Tarca AL, Chaemsaithong P, Xu Z, Fitzgerald W (2015). Evidence of perturbations of the cytokine network in preterm labor. Am J Obstet Gynecol.

[22] Strauss JF, Romero R, Gomez-Lopez N, Haymond-Thornburg H, Modi BP, Teves ME (2018). Spontaneous preterm birth: advances toward the discovery of genetic predisposition. Am J Obstet Gynecol.

[23] Romero R, Espinoza J, Gonçalves LF, Kusanovic JP, Friel L, Hassan S (2007). The role of inflammation and infection in preterm birth. Semin Reprod Med.

[24] Nhan-Chang C-L, Romero R, Tarca AL, Mittal P, Kusanovic JP, Erez O (2010). Characterization of the transcriptome of chorioamniotic membranes at the site of rupture in spontaneous labor at term. Am J Obstet Gynecol.

[25] Challis JR, Lockwood CJ, Myatt L, Norman JE, Strauss JF, Petraglia F. Inflammation and pregnancy. In: Reproductive Sciences. 2009. p. 206–15. 10.1177/1933719108329095 19208789

[26] Vrachnis N, Vitoratos N, Iliodromiti Z, Sifakis S, Deligeoroglou E, Creatsas G (2010). Intrauterine inflammation and preterm delivery. Ann N Y Acad Sci.

[27] De Mucio B, Abalos E, Cuesta C, Carroli G, Serruya S, Giordano D (2016). Maternal near miss and predictive ability of potentially life-threatening conditions at selected maternity hospitals in Latin America. Reprod Health.

[28] Leek JT, Scharpf RB, Bravo HC, Simcha D, Langmead B, Evan Johnson W (2010). Tackling the widespread and critical impact of batch effects in high-throughput data. Nat Rev Genet.

[29] Bolger AM, Lohse M, Usadel B (2014). Trimmomatic: a flexible trimmer for Illumina sequence data. Bioinformatics.

[30] Andrews S. FastQC: A quality control tool for high throughput sequence data. Http://Www.Bioinformatics.Babraham.Ac.Uk/Projects/Fastqc/. 2010. doi:citeulike-article-id:11583827.

[31] Kim D, Pertea G, Trapnell C, Pimentel H, Kelley R, Salzberg SL. TopHat2: Accurate alignment of transcriptomes in the presence of insertions, deletions and gene fusions. Genome Biol. 2013;14. 10.1186/gb-2013-14-4-r36. 10.1186/gb-2013-14-4-r36 PMC4053844 23618408

[32] Aken BL, Ayling S, Barrell D, Clarke L, Curwen V, Fairley S, et al. The Ensembl gene annotation system. Database . 2016;2016. 10.1093/database/baw093. 10.1093/database/baw093 PMC4919035 27337980

[33] Love MI, Huber W, Anders S. Moderated estimation of fold change and dispersion for RNA-seq data with DESeq2. Genome Biol. 2014;15. 10.1186/s13059-014-0550-8. 10.1186/s13059-014-0550-8 PMC4302049 25516281

[34] Anders S, Huber W. Differential expression analysis for sequence count data. Genome Biol. 2010;11. 10.1186/gb-2010-11-10-r106. 10.1186/gb-2010-11-10-r106 PMC3218662 20979621

[35] Robinson MD, McCarthy DJ, Smyth GK (2010). edgeR: a Bioconductor package for differential expression analysis of digital gene expression data. Bioinformatics.

[36] Trapnell C, Hendrickson DG, Sauvageau M, Goff L, Rinn JL, Pachter L (2013). Differential analysis of gene regulation at transcript resolution with RNA-seq. Nat Biotechnol.

[37] Benjamini Y, Hochberg Y (1995). Controlling the false discovery rate: a practical and powerful approach to multiple testing. J R Stat Soc B..

[38] Luo W, Friedman MS, Shedden K, Hankenson KD, Woolf PJ. GAGE: Generally applicable gene set enrichment for pathway analysis. BMC Bioinformatics. 2009;10. 10.1186/1471-2105-10-161. 10.1186/1471-2105-10-161 PMC2696452 19473525

[39] Young MD, Wakefield MJ, Smyth GK, Oshlack A. Gene ontology analysis for RNA-seq: accounting for selection bias. Genome Biol. 2010;11. 10.1186/gb-2010-11-2-r14. 10.1186/gb-2010-11-2-r14 PMC2872874 20132535

[40] Durinck S, Spellman PT, Birney E, Huber W (2009). Mapping Identifiers for the Integration of Genomic Datasets with the R/Bioconductor package biomaRt. Nat Protoc.

[41] Uzun A, Laliberte A, Parker J, Andrew C, Winterrowd E, Sharma S (2012). dbPTB: a database for preterm birth. Database.

[42] Vora B, Wang A, Kosti I, Huang H, Paranjpe I, Woodruff TJ (2018). Meta-Analysis of Maternal and Fetal Transcriptomic Data Elucidates the Role of Adaptive and Innate Immunity in Preterm Birth. Front Immunol.

[43] Bukowski R, Sadovsky Y, Goodarzi H, Zhang H, Biggio JR, Varner M (2017). Onset of human preterm and term birth is related to unique inflammatory transcriptome profiles at the maternal fetal interface. PeerJ.

[44] Heng YJ, Pennell CE, Chua HN, Perkins JE, Lye SJ (2014). Whole blood gene expression profile associated with spontaneous preterm birth in women with threatened preterm labor. PLoS One.

[45] Ngo TTM, Moufarrej MN, Rasmussen M-LH, Camunas-Soler J, Pan W, Okamoto J (2018). Noninvasive blood tests for fetal development predict gestational age and preterm delivery. Science.

[46] Lee J, Romero R, Chaiworapongsa T, Dong Z, Tarca AL, Xu Y (2013). Characterization of the fetal blood transcriptome and proteome in maternal anti-fetal rejection: evidence of a distinct and novel type of human fetal systemic inflammatory response. Am J Reprod Immunol.

[47] Livak KJ, Schmittgen TD (2001). Analysis of relative gene expression data using real-time quantitative PCR and the 2- ΔΔCT method. Methods.

[48] Wickham H. ggplot2: Elegant Graphics for Data Analysis, 2nd edn. New York: Springer; 2016. 10.1007/978-3-319-24277-4_5.

[49] Haddad R, Tromp G, Kuivaniemi H, Chaiworapongsa T, Kim YM, Mazor M (2006). Human spontaneous labor without histologic chorioamnionitis is characterized by an acute inflammation gene expression signature. Am J Obstet Gynecol.

[50] Stephen GL, Lui S, Hamilton SA, Tower CL, Harris LK, Stevens A, et al. Transcriptomic Profiling of Human Choriodecidua During Term Labor: Inflammation as a Key Driver of Labor. Am J Reprod Immunol. 2015. 10.1111/aji.12328. 10.1111/aji.12328 25283845

[51] El-Azzamy H, Balogh A, Romero R, Xu Y, LaJeunesse C, Plazyo O (2017). Characteristic Changes in Decidual Gene Expression Signature in Spontaneous Term Parturition. J Pathol Transl Med.

[52] Hamilton SA, Tower CL, Jones RL (2013). Identification of chemokines associated with the recruitment of decidual leukocytes in human labour: potential novel targets for preterm labour. PLoS One.

[53] de Jong E, Hancock DG, Wells C, Richmond P, Simmer K, Burgner D, et al. Exposure to chorioamnionitis alters the monocyte transcriptional response to the neonatal pathogen *Staphylococcus epidermidis*. Immunol Cell Biol. 2018. 10.1111/imcb.12037. 10.1111/imcb.12037 29533486

[54] Chim S, Wong K, Chung C, Lam S, Kwok J, Lai C-Y (2017). Systematic Selection of Reference Genes for the Normalization of Circulating RNA Transcripts in Pregnant Women Based on RNA-Seq Data. Int J Mol Sci.

[55] Migale R, MacIntyre DA, Cacciatore S, Lee YS, Hagberg H, Herbert BR (2016). Modeling hormonal and inflammatory contributions to preterm and term labor using uterine temporal transcriptomics. BMC Med.

[56] Willcockson AR, Nandu T, Liu C-L, Nallasamy S, Kraus WL, Mahendroo M (2018). Transcriptome signature identifies distinct cervical pathways induced in lipopolysaccharide-mediated preterm birth. Biol Reprod.

[57] Saben J, Zhong Y, McKelvey S, Dajani NK, Andres A, Badger TM (2014). A comprehensive analysis of the human placenta transcriptome. Placenta.

[58] Blencowe H, Cousens S, Oestergaard MZ, Chou D, Moller A-B, Narwal R (2012). National, regional, and worldwide estimates of preterm birth rates in the year 2010 with time trends since 1990 for selected countries: a systematic analysis and implications. Lancet.

[59] Challis JR, Lockwood CJ, Myatt L, Norman JE, Strauss JF, Petraglia F (2009). Inflammation and pregnancy. Reprod Sci.

[60] Behrman RE, Butler AS (2007). Preterm birth: Causes, consequences, and prevention.

[61] Vitoratos N, Papadias K, Makrakis E, Christodoulakos G, Panoulis K, Creatsas G (2006). Association between serum tumor necrosis factor-alpha and corticotropin-releasing hormone levels in women with preterm labor. J Obstet Gynaecol Res.

[62] Flood K, Malone FD (2012). Prevention of preterm birth. Semin Fetal Neonatal Med.

[63] Korebrits C, Ramirez MM, Watson L, Brinkman E, Bocking AD, Challis JR (1998). Maternal corticotropin-releasing hormone is increased with impending preterm birth. J Clin Endocrinol Metab.

[64] Watari M, Watari H, DiSanto ME, Chacko S, Shi G-P, Strauss JF (1999). Pro-Inflammatory Cytokines Induce Expression of Matrix-Metabolizing Enzymes in Human Cervical Smooth Muscle Cells. Am J Pathol.

[65] Heng YJ, Liong S, Permezel M, Rice GE, Di Quinzio MKW, Georgiou HM (2014). The interplay of the interleukin 1 system in pregnancy and labor. Reprod Sci.

[66] Watari M, Watari H, Strauss J (2000). Lipopolysaccharide and pro-inflammatory cytokines induce expression of matrix metabolizing enzymes in human cervical smooth muscle cells-implication in the mechanism of cervical ripening. Int J Gynaecol Obstet.

[67] Franchi L, Eigenbrod T, Muñoz-Planillo R, Nuñez G (2009). The inflammasome: a caspase-1-activation platform that regulates immune responses and disease pathogenesis. Nat Immunol.

[68] Shao B-Z, Xu Z-Q, Han B-Z, Su D-F, Liu C (2015). NLRP3 inflammasome and its inhibitors: a review. Front Pharmacol.

[69] van de Veerdonk FL, Netea MG, Dinarello CA, Joosten LAB. Inflammasome activation and IL-1β and IL-18 processing during infection. Trends Immunol. 2011;32:110-16. 10.1016/j.it.2011.01.003 21333600

[70] Petrilli V, Papin S, Tschopp J (2005). The inflammasome. Curr Biol.

[71] Jin C, Flavell RA (2010). Molecular mechanism of NLRP3 inflammasome activation. J Clin Immunol.

[72] Gotsch F, Romero R, Chaiworapongsa T, Erez O, Vaisbuch E, Espinoza J (2008). Evidence of the involvement of caspase-1 under physiologic and pathologic cellular stress during human pregnancy: a link between the inflammasome and parturition. J Matern Fetal Neonatal Med.

[73] Gomez-Lopez N, Romero R, Xu Y, Plazyo O, Unkel R, Leng Y (2017). A Role for the Inflammasome in Spontaneous Preterm Labor With Acute Histologic Chorioamnionitis. Reprod Sci.

[74] Plazyo O, Romero R, Unkel R, Balancio A, Mial TN, Xu Y (2016). HMGB1 Induces an Inflammatory Response in the Chorioamniotic Membranes That Is Partially Mediated by the Inflammasome. Biol Reprod.

[75] Romero R, Xu Y, Plazyo O, Chaemsaithong P, Chaiworapongsa T, Unkel R (2018). A Role for the Inflammasome in Spontaneous Labor at Term. Am J Reprod Immunol.

[76] Panaitescu B, Romero R, Gomez-Lopez N, Xu Y, Leng Y, Maymon E, et al. In vivo evidence of inflammasome activation during spontaneous labor at term. J Matern Neonatal Med. 2019;32:1978–91. 10.1080/14767058.2017.1422714. 10.1080/14767058.2017.1422714 PMC6050140 29295667

[77] Pacora P, Romero R, Maymon E, Gervasi MT, Gomez R, Edwin SS (2000). Participation of the novel cytokine interleukin 18 in the host response to intra-amniotic infection. Am J Obstet Gynecol.

[78] Holt R, Timmons BC, Akgul Y, Akins ML, Mahendroo M (2011). The molecular mechanisms of cervical ripening differ between term and preterm birth. Endocrinology.

[79] Maymon E, Romero R, Pacora P, Gomez R, Athayde N, Edwin S (2000). Human neutrophil collagenase (matrix metalloproteinase 8) in parturition, premature rupture of the membranes, and intrauterine infection. Am J Obstet Gynecol.

[80] Kim SM, Romero R, Lee J, Chaemsaithong P, Lee M-W, Chaiyasit N (2016). About one-half of early spontaneous preterm deliveries can be identified by a rapid matrix metalloproteinase-8 (MMP-8) bedside test at the time of mid-trimester genetic amniocentesis. J Matern Fetal Neonatal Med.

[81] Arechavaleta-Velasco F, Marciano D, Díaz-Cueto L, Parry S (2004). Matrix metalloproteinase-8 is expressed in human chorion during labor. Am J Obstet Gynecol.

[82] Witkin SS (2015). The vaginal microbiome, vaginal anti-microbial defence mechanisms and the clinical challenge of reducing infection-related preterm birth. BJOG.

[83] Farr M, Strübe J, Geppert HG, Kocourek A, Mahne M, Tschesche H (2000). Pregnancy-associated plasma protein-E (PAPP-E). Biochim Biophys Acta.

[84] Chim SSC, Lee WS, Ting YH, Chan OK, Lee SWY, Leung TY (2012). Systematic identification of spontaneous preterm birth-associated RNA transcripts in maternal plasma. PLoS One.

[85] Winn VD, Haimov-Kochman R, Paquet AC, Yang YJ, Madhusudhan MS, Gormley M (2007). Gene expression profiling of the human maternal-fetal interface reveals dramatic changes between midgestation and term. Endocrinology.

[86] Partl JZ, Karin V, Skrtic A, Nikuseva-Martic T, Serman A, Curkovic-Perica M, et al. Negative regulators of Wnt signaling pathway SFRP1 and SFRP3 expression in preterm and term pathologic placentas. J Matern Neonatal Med. 2018;31:2971–9. 10.1080/14767058.2017.1359830. 10.1080/14767058.2017.1359830 28738713

[87] Novakovic B, Fournier T, Harris LK, James J, Roberts CT, Yong HEJ (2017). Increased methylation and decreased expression of homeobox genes TLX1, HOXA10 and DLX5 in human placenta are associated with trophoblast differentiation. Sci Rep.

[88] Zadora J, Singh M, Herse F, Przybyl L, Haase N, Golic M (2017). Disturbed Placental Imprinting in Preeclampsia Leads to Altered Expression of DLX5, a Human-Specific Early Trophoblast Marker. Circulation.

[89] Eidem HR, Rinker DC, Ackerman WE, Buhimschi IA, Buhimschi CS, Dunn-Fletcher C (2016). Comparing human and macaque placental transcriptomes to disentangle preterm birth pathology from gestational age effects. Placenta.

[90] Davidson LM, Coward K (2016). Molecular mechanisms of membrane interaction at implantation. Birth Defects Res C Embryo Today.

[91] Wray S, Burdyga T, Noble D, Noble K, Borysova L, Arrowsmith S (2015). Progress in understanding electro-mechanical signalling in the myometrium. Acta Physiol.

[92] Buxton ILO, Heyman N, Wu Y-Y, Barnett S, Ulrich C (2011). A role of stretch-activated potassium currents in the regulation of uterine smooth muscle contraction. Acta Pharmacol Sin.

[93] Vaeth M, Feske S (2018). Ion channelopathies of the immune system. Curr Opin Immunol.

[94] Gekas C, Dieterlen-Lièvre F, Orkin SH, Mikkola HKA (2005). The placenta is a niche for hematopoietic stem cells. Dev Cell.

[95] Khodadi E, Shahrabi S, Shahjahani M, Azandeh S, Saki N (2016). Role of stem cell factor in the placental niche. Cell Tissue Res.

[96] Alvarez-Silva M, Belo-Diabangouaya P, Salaün J, Dieterlen-Lièvre F (2003). Mouse placenta is a major hematopoietic organ. Development.

[97] Ottersbach K, Dzierzak E (2009). Analysis of the mouse placenta as a hematopoietic stem cell niche. Methods Mol Biol.

[98] Kuchma MD, Kyryk VM, Svitina HM, Shablii YM, Lukash LL, Lobyntseva GS (2015). Comparative Analysis of the Hematopoietic Progenitor Cells from Placenta, Cord Blood, and Fetal Liver, Based on Their Immunophenotype. Biomed Res Int.

[99] Mattern A, Zellmann T, Beck-Sickinger AG (2014). Processing, signaling, and physiological function of chemerin. IUBMB Life.

[100] Kobayashi Y (2008). The role of chemokines in neutrophil biology. Front Biosci.

[101] Mahmud ZA, Jenkins L, Ulven T, Labéguère F, Gosmini R, De Vos S (2017). Three classes of ligands each bind to distinct sites on the orphan G protein-coupled receptor GPR84. Sci Rep.

[102] Presicce P, Park C-W, Senthamaraikannan P, Bhattacharyya S, Jackson C, Kong F, et al. IL-1 signaling mediates intrauterine inflammation and chorio-decidua neutrophil recruitment and activation. JCI Insight. 2018;3. 10.1172/jci.insight.98306. 10.1172/jci.insight.98306 PMC5926925 29563340

[103] Uusküla L, Männik J, Rull K, Minajeva A, Kõks S, Vaas P (2012). Mid-gestational gene expression profile in placenta and link to pregnancy complications. PLoS One.

[104] Zhu YX, Benn S, Li ZH, Wei E, Masih-Khan E, Trieu Y (2004). The SH3-SAM adaptor HACS1 is up-regulated in B cell activation signaling cascades. J Exp Med.

[105] Rahmioglu N, Drong AW, Lockstone H, Tapmeier T, Hellner K, Saare M (2017). Variability of genome-wide DNA methylation and mRNA expression profiles in reproductive and endocrine disease related tissues. Epigenetics.

[106] Ackerman WE 4th, Buhimschi IA, Brubaker D, Maxwell S, Rood KM, Chance MR, et al. Integrated microRNA and mRNA network analysis of the human myometrial transcriptome in the transition from quiescence to labor. Biol Reprod. 2018. 10.1093/biolre/ioy040. 10.1093/biolre/ioy040 PMC5991200 29447339

[107] Menon R, Richardson LS (2017). Preterm prelabor rupture of the membranes: A disease of the fetal membranes. Semin Perinatol.

[108] Plunkett J, Muglia LJ (2008). Genetic contributions to preterm birth: implications from epidemiological and genetic association studies. Ann Med.

[109] Plunkett J, Doniger S, Orabona G, Morgan T, Haataja R, Hallman M (2011). An Evolutionary Genomic Approach to Identify Genes Involved in Human Birth Timing. PLoS Genet.

[110] Crider KS, Whitehead N, Buus RM (2005). Genetic variation associated with preterm birth: A HuGE review. Genet Med.

[111] Romero R, Mazaki-Tovi S, Vaisbuch E, Kusanovic JP, Chaiworapongsa T, Gomez R (2010). Metabolomics in premature labor: a novel approach to identify patients at risk for preterm delivery. J Matern Fetal Neonatal Med.

[112] Kim J. Identification of genes contributing to preterm birth: insights from genetic, transcriptomic, and epigenetic analyses. PhD thesis, University of Iowa, 2012. 10.17077/etd.p3zuvpa3.

[113] Sans M, Salzano FM, Chakraborty R (1997). Historical genetics in Uruguay: estimates of biological origins and their problems. Hum Biol.

[114] Hidalgo PC, Bengochea M, Abilleira D, Cabrera A, Alvarez I (2005). Genetic Admixture Estimate in the Uruguayan Population Based on the Loci LDLR, GYPA, HBGG, GC and D7S8. Int J Hum Genet.

[115] Yang E, Wang G, Yang J, Zhou B, Tian Y, Cai JJ (2016). Epistasis and destabilizing mutations shape gene expression variability in humans via distinct modes of action. Hum Mol Genet.

[116] Lappalainen T, Sammeth M, Friedländer MR, PAC ‘t h, Monlong J, Rivas MA (2013). Transcriptome and genome sequencing uncovers functional variation in humans. Nature..

[117] Cappetta M, Berdasco M, Hochmann J, Bonilla C, Sans M, Hidalgo PC (2015). Effect of genetic ancestry on leukocyte global DNA methylation in cancer patients. BMC Cancer.

