# Supplementary figures and images for: Transcriptomic analysis of fetal membranes reveals pathways involved in preterm birth
Source: BMC Med Genomics. 2019 Apr 1;12:53. doi: 10.1186/s12920-019-0498-3 (PMC6444860; doi:10.1186/s12920-019-0498-3)

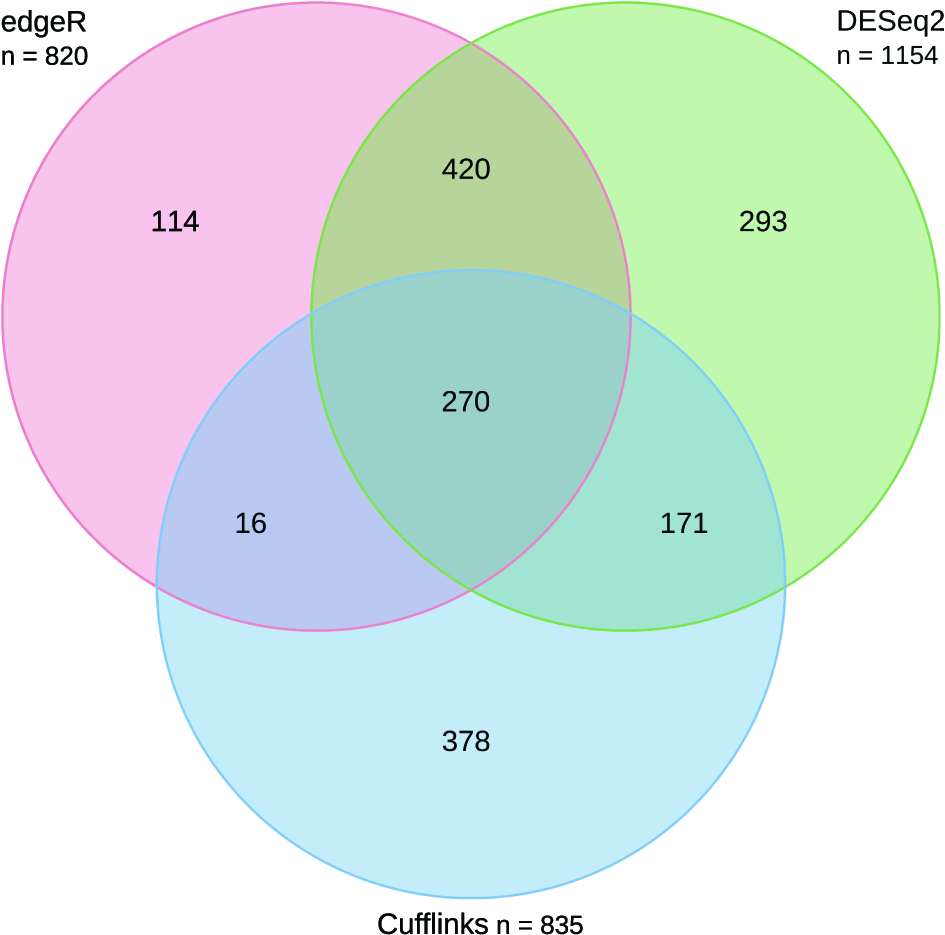

Supplement: Supplementary file 4 — Figure S1. Differentially expressed genes. Venn diagram showing the number of differentially expressed genes identified by each of the three methods employed, when considering FDR < 0.05. (TIF 906 kb) [file 12920_2019_498_MOESM4_ESM.tif]
